# Interference Analysis for mmWave Automotive Radar Considering Blockage Effect

**DOI:** 10.3390/s21123962

**Published:** 2021-06-08

**Authors:** Liping Kui, Sai Huang, Zhiyong Feng

**Affiliations:** School of Information and Communication Engineering, Beijing University of Posts and Telecommunications, Beijing 100876, China; kuiliping@bupt.edu.cn (L.K.); fengzy@bupt.edu.cn (Z.F.)

**Keywords:** mmWave automotive radars, stochastic geometry, interference analysis, blockage modeling, radar ranging performance

## Abstract

Due to the increasing number of vehicles equipped with millimeter wave (mmWave) radars, interference among automotive radars is becoming a major issue. This paper explores the automotive radar interference in both two-lane and multi-lane scenarios using stochastic geometry. We derive closed-form expressions for mean and variance of interference power considering directional antenna with constant and Gaussian decaying gains. In view of the sensitivity of mmWave radar signals to the blockages, we propose a blockage model including partially and completely blocking, and then calculate the effective number of the interferers. By means of modeling randomness for interferers and blockages as Poisson point process, we characterize the statistics of radar interference under different conditions. We further utilize the interference characterization to estimate the successful ranging probability of automotive radars. These theoretical analyses are verified by using Monte Carlo simulations. The results show that the increasing interfering density and ranging distance largely degrade the radar detection performance, whereas the interference levels decrease as blockage intensity increases.

## 1. Introduction

Millimeter wave (mmWave) radars are being widely used in modern vehicles for supporting the applications of advanced driver assistance systems (ADAS) and autonomous driving, thus providing comfort and safety functions, such as adaptive cruise control (ACC), blind spot detection (BSD), lane change assist (LCA), emergency braking and collision avoidance [1,2]. To date, most automotive radar transmissions are uncoordinated, meaning that automotive radars operating in the same frequency band may interfere with each other when transmitting signals at the same time and in the same area [3,4]. Such mutual interference can decrease the radar detection performance, or lead to ghost targets. In addition, serious interference between automotive radars will incur risks for future automated driving in addition to reducing sensitivity. Therefore, it is necessary to analyze the levels of automotive radar interference in common road scenarios. Moreover, interference analysis is beneficial to design effective countermeasures minimizing interference with high traffic density and a rising percentage of vehicles equipped with radars.

There have been many studies analyzing the interference of mmWave automotive radars. In [5], mutual interference between two automotive FMCW radars operating in overlapping bands was investigated under different weather conditions, and the main effect factors related to interference levels have also been thoroughly discussed. Goppelt et al. first qualitatively analyzed the interference between FMCW radars and pulsed radars in the simple mutual interference scenario [6], and then enhanced the previous qualitative analysis by the quantitative calculation of the interference power [7]. Since the automotive radar waveforms are not regulated, Bourdoux et al. [8] graphically illustrated the phenomenology of the mutual interference among the four possible interferer–victim pairs of FMCW and PMCW radars. Similarly, [9] explored the mutual interference for automotive chirp sequence (CS) radars, and showed that interference effects between CS and FMCW radars are equivalent.

Although the above studies deeply analyzed and investigated the mutual interference for most automotive radar waveforms in simple two-node topologies, the work on quantifying the interference problem in realistic traffic scenarios is still limited. In [10], a simulation model based on ray-tracing was devised to predict the received interference power at an automotive victim radar for different traffic situations. Furthermore, the stochastic geometry method was leveraged to evaluate interference characterization in automotive radar network [11,12,13,14,15,16]. Al-Hourani et al. [11] formulated an analytic framework characterizing the radar interference in terms of its cumulative distribution function and mean value, where locations of vehicular nodes on each lane are modeled as a Poisson point process (PPP) and a regular lattice. The authors also revealed that the models of vehicle distribution have a limited effect on the interference statistics. An analytical approach was proposed in [12] to study the effect of mutual interference in a planar network of pulsed-radar devices. Specifically, the authors-derived compact closed-form expressions for the radar detection performance with different path-loss exponents in the no-fading and Rayleigh-fading cases. The maximum and the mean values of the total interference power are calculated for only a two-lane highway in [13]. The interference analysis is extended to a multi-lane road in [14], and the aggregated interference from interfering radars depends on the properties of the individual radars as well as the placement of vehicles on the road. The work in [15] examined the extent of automotive radar interference including incident interference and reflected interference. Meanwhile, the fluctuation of the target radar cross section (RCS) is considered to precisely analyze the radar ranging performance by using Swerling I model and Chi-square model [16].

However, in the above studies, the gain of directional antenna in the main lobe is always assumed to be a constant, regardless of the locations of interferers relative to the victim, which is equivalent to the case of omnidirectional radiation pattern or completely aligned main beam. Employing such a flat main lobe, the results of the interference analysis may be significantly larger than actual ones. In addition, these studies do not consider the effects of blockage on radar interference among multiple vehicles, ultimately resulting in exaggerated interference. The authors in [17] derived closed-form expressions of the mean interference power for automotive radar by approximating the antenna radiation pattern with Gaussian waveforms. This gain of directional antenna was represented by a Gaussian function. Nevertheless, this approach of interference analysis only applies to the no-fading case with the path-loss exponent of 2 in the absence of blockages.

As the directional beam of the mmWave signal is quite sensitive to blocking, many works for analyzing the impact of blockage on interference in mmWave cellular networks have been carried out [18,19,20,21,22]. Unlike these networks where the wireless nodes are distributed uniformly over the plane, the locations of vehicular nodes are restricted to roadways. Therefore, these blocking models can not directly be used for analyzing interference in an automotive radar network. In urban vehicular scenarios, a model of vehicle-body blockage is proposed in [23], where the line-of-sight (LOS) path between the communicating nodes is occluded by vehicles. Since the height of the transmitter (TX) and receiver (RX) is not the same, the height of blockage is usually taken as an important parameter in calculating blockage probability. However, mmWave automotive radars, e.g., the long-range radars (LRRs), are typically mounted on the front of the vehicles at same height. The blockage model relating to height of the blockers is not applicable to characterize the blockage effect on automotive radar interference. Boban et al. [24] performed vehicle-to-vehicle (V2V) channel measurements in urban and highway scenarios, and then explored the impact of the vehicle blocker size and position on the received power. Aiming at automotive radar interference, measurements of the blockage effect face many problems [10], especially since higher radar penetration rate (RPR) is not yet present, which will cause insignificant statistical result. According to the above analyses, at present, few studies are concerned with the influence of blocking on automotive radar interference.

In this paper, we propose an analytical framework of interference power for automotive radars in both two-lane and multi-lane scenarios. Considering different directional angles between interferers and victim, we adopt a directional antenna whose radiation pattern in the main lobe is modeled by a Gaussian decaying function. The influence of blockage on the interference signal was taken into consideration to evaluate the levels of aggregated interference among multiple radars. Utilizing the stochastic geometry method to characterize the randomness of locations and number of vehicles and blockages, we then derive the signal-to-interference-and-noise ratio (SINR)-based successful ranging probability of automotive radar. Our main contributions can be summarized as follows.

- We derive the mean and variance of the aggregated interference power of the victim radar equipped with constant directional gain antenna, under no-fading, Rayleigh-fading and Nakagami-fading cases with any path-loss exponent;
- Employing the directional antenna with Gaussian attenuation gain in the main lobe, we first derive closed-form expressions of the mean and variance of interference in the two-lane scenario and then extend the work to multi-lane one;
- We propose a blockage model considering both complete and partial blocking, and calculate the corresponding blockage probabilities. The expected number of active interferers is obtained by computing its probability-generating function (PGF). Based on this blockage model, we compare the levels of automotive radar interference with and without considering blockage;
- The above theoretical analysis and derivation are verified by using Monte Carlo simulations. We validate the supposed distribution model of aggregated interference, and then provide radar performance estimation based on the interference statistics.

The rest of this paper is organized as follows. The system and signal models are described in Section 2. The proposed interference analysis framework is constructed for two-lane scenarios in Section 3. Section 4 extends the work characterizing the interference level to multi-lane scenarios. Automotive radar performance based on SINR is formulated in Section 5. In Section 6, numerous simulation results are presented and analyzed. Finally, conclusions are drawn in Section 7.

## 2. System Model

**Figure 1:**
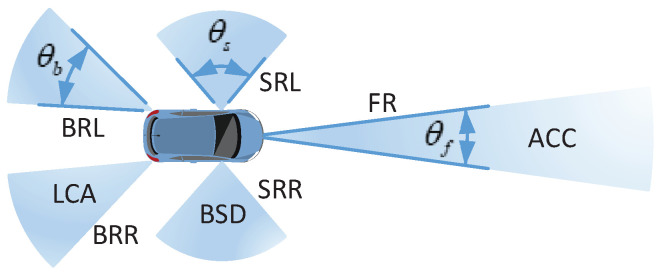
Three typical kinds of radars used for a modern vehicle.

The common setup of a modern vehicle is a forward looking radar (FR) to enable ACC, two sideward left and right looking radars (SRL, SRR) and two backward left and right looking radars (BRL, BRR) to provide BSD and LCA, respectively, [1,2], as shown in Figure 1. The FR typically adopts a long-range radar (LRR) for applications where a narrow-beam forward-looking view is required. The SR and BR severally employ the short-range radar (SRR) and the medium-range radar (MRR) to implement their functions. These three types of radars are usually deployed over different frequency ranges [1,25], therefore, there is little interference between them. This paper mainly focuses on the interference analysis of the FRs, both in two-lane and multiple-lane scenarios.

We consider a population of FMCW mmWave automotive radars, modeling the geometry distribution of vehicular nodes as a homogeneous one-dimensional Poisson point process (1-D PPP) with intensity ρ on each lane. Thus, the distribution of vehicles on each lane is independent and identical with that on other lanes. Without loss of generality, the victim radar is mounted on the front of the bumper of a vehicle located at the origin and called the typical vehicle. Assume that the vehicles are stationary during the small radar detection period. In other words, we analyze the automotive interference in a temporal snapshot of the road traffic. Suppose that parameter $\varepsilon \in \left[0,1\right]$ represents the probability that the vehicle radar uses the same frequency with the victim radar at same time in the same area, also known as the probability of spectrum resource collision [11]. Let ρ denote the vehicle density, then $\lambda_{I}=\varepsilon \rho$ is the intensity of interferers on each lane.

The typical radar will receive two kinds of signals composed of target echo signals and interference signals except for the noise signal. The target echo signal is the desired signal for the typical receiver, which is reflected by the target being tracked and primarily used to realize ranging and positioning. Most of interference signals come from the automotive radars operating at the same frequency with the typical radar. In this paper, we mainly investigate the direct incident interference caused by the oncoming vehicles moving in the opposite direction, where the interfering radars have an overlapping beam with the typical radar.

For a target vehicle with a radar cross section (RCS) $\sigma_{c}$, the desired echo-power $P_{echo}$ received at the typical radar receiver can be calculated by

(1) $$P_{echo}=\frac{P_{t}G_{t}^{v}G_{r}^{v}\lambda^{2}\sigma_{c}h_{0}}{{\left(4\pi \right)}^{3}d_{0}^{2\alpha }}=\frac{P_{t}G_{0}^{2}\lambda^{2}\sigma_{c}h_{0}}{{\left(4\pi \right)}^{3}d_{0}^{2\alpha }}=A_{0}h_{0}d_{0}^{-2\alpha }\;,$$

where $P_{t}$ is the radar transmit power, $h_{0}$ represents the channel fading gain. $G_{t}^{v}$, $G_{r}^{v}$ and $G_{0}$ are the transmit antenna gain, receive antenna gain and maximum antenna gain, respectively, for the above situation $G_{t}^{v}=G_{r}^{v}=G_{0}$. α is the path-loss exponent, and $d_{0}$ denotes the range from the target to the typical vehicle. The coefficient A0=PtG02λ2σcPtG02λ2σc4π34π3 and the wavelength λ=ccfcfc, where *c* is speed of light and $f_{c}$ is the center frequency.

We assume all automotive radars have the same transmit power and the interference effects caused by these radars on the victim are independent of each other. The interference power of the *i*th interferer is given by

(2) $$P_{I_{i}}=\frac{P_{t}G_{t}^{i}G_{r}^{vi}\lambda^{2}h_{i}}{{\left(4\pi \right)}^{2}r_{i}^{\alpha }}=\frac{P_{t}G_{i}^{2}\lambda^{2}}{{\left(4\pi \right)}^{2}}h_{i}r_{i}^{-\alpha }\;,$$

where $h_{i}$ is channel gain, $r_{i}=\sqrt{d_{i}^{2}+L_{n}^{2}}$ refers to the Euclidean distance between the *i*th interferer and the typical radar, in which $d_{i}$ and $L_{n}$ are the horizontal and vertical distances, respectively. The antenna gain $G_{t}^{i}$ of the interfering radar is equal to that of the victim radar $G_{r}^{vi}$, namely $G_{t}^{i}=G_{r}^{vi}=G_{i}$. The aggregated interference power of the typical radar can be written as

(3) $$P_{I_{agg}}=\sum_{i=1}^{k}P_{I_{i}}=\sum_{i=1}^{k}P_{t}{\left(\frac{\lambda }{4\pi }\right)}^{2}G_{i}^{2}h_{i}r_{i}^{-\alpha }\;.$$

## 3. Automotive Radar Interference Analysis in Two-Lane Scenarios

**Figure 2:**
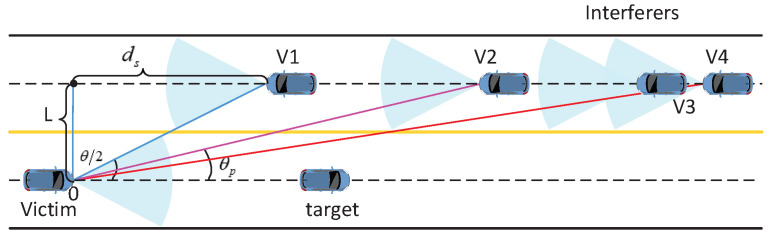
The interference between automotive radars in two-lane scenario.

We consider a mutual interference scenario with direct interference from the oncoming vehicles driving in the opposite direction to the typical vehicle (victim), as illustrated in Figure 2, where V1, V2, V3, and V4 indicate interfering vehicles, *L* is lane spacing, θ and $\theta_{p}$ express the beamwidth of antenna and directional angle between victim and interferer, respectively. The width of green belts and the central median among the road are not taken into consideration. The minimum horizontal distance $d_{s}$ between the victim and interferers is given by ds=LLtanθθ22tanθθ22. The interfering vehicles must be located beyond the minimum distance. V1 happens to be located at the extreme distance and its interference power is 0.

In this section, we first derive the general expressions of the mean and the variance of the aggregated interference power with constant antenna gain for no-fading, Nakagmi fading and Rayleigh fading cases with different path-loss exponents. Subsequently, employing the directional antenna with Gaussian waveform gain, we derive the closed-form expressions for statistical characteristics of interference power. Finally, the above analysis methodology and work are extended to the considering blockage case.

### 3.1. Interference Modeling and Methodology

The mean interference power can be solved in two ways, either by utilizing the Campbell’s theorem [26], or by the derivation of the mean definition of aggregated interference directly [27]. We obtain the mean and variance of aggregated interference by using the latter method. Since vehicle nodes are modeled by 1-D PPP, the number *N* of interfering vehicles located at the interval $\left[d_{s},d_{e}\right]$ follows Poisson distribution with mean and variance:

(4) $$E\left[N\right]=\lambda_{I}\left(d_{e}-d_{s}\right),\;Var\left[N\right]=E\left[N\right]\;,$$

in which $d_{e}$ denotes the maximum range of interference. The horizontal distance to the typical radar receiver (the origin 0), denoted as a random variable *D*, has the same probability density fuction (PDF) for any interferer *i*:

(5) $$f_{D}\left(d\right)=\frac{1}{d_{e}-d_{s}}\;,\;d_{s}<d<d_{e}\;.$$

We start the interference analysis applying a flat-top directional antenna, which has constant gain within its beamwidth and zero gain outside the beamwidth, like in the literature [11,12,13,14,15,16]. Among these, the maximum antenna gain is usually taken as the transmit and receive antenna gain in [11,12]. Therefore, the mean interference power of the *i*th interfering radar with a constant antenna gain is expressed as

(6) $$E\left[P_{I_{i}}\right]=E\left[\frac{P_{t}G_{0}^{2}\lambda^{2}}{{\left(4\pi \right)}^{2}}h_{i}r_{i}^{-\alpha }\right]=E\left[Ah_{i}r_{i}^{-\alpha }\right]\;=\;AE\left[h_{i}\right]E\left[r_{i}^{-\alpha }\right]\;,$$

where the channel gain $h_{i}$ and the distance $r_{i}$ are independent, the coefficient A=PtG02λ2PtG02λ24π24π2.

According to Figure 2, the distance $r_{i}=\sqrt{d_{i}^{2}+L^{2}}$ and $d_{i}∼U\left(d_{s},d_{e}\right)$, then mean of the random variable $r_{i}^{-\alpha }$ is readily computed to be: -4.6cm0cm

(7) $$E\left[r_{i}^{-\alpha }\right]\;=\;\int_{d_{s}}^{d_{e}}\;r_{i}^{-\alpha }f_{D}\left(t\right)dt\;=\;\frac{1}{d_{e}-d_{s}}\int_{d_{s}}^{d_{e}}\;\frac{1}{{\left(\sqrt{t^{2}+L^{2}}\right)}^{\alpha }}dt\;=\;\frac{1}{d_{e}-d_{s}}{\left.\frac{t{\left(\frac{t^{2}}{L^{2}}+1\right)}^{\frac{\alpha }{2}}\cdot {}_{2}F_{1}\left(\frac{1}{2},\frac{\alpha }{2};\frac{3}{2};-\frac{t^{2}}{L^{2}}\right)}{{\left(t^{2}+L^{2}\right)}^{\frac{\alpha }{2}}}\right|}_{d_{s}}^{d_{e}}\;,$$

where ${}_{2}F_{1}\left(\cdot \;,\;\cdot \;;\;\;\cdot \;;\;\cdot \right)$ is the hypergeometric function.

The aggregated interference of the typical radar depends on the interference levels of the individual radars, and the effects from these interfering radars are independent. Hence, the mean value of the aggregated interference at the typical vehicle can be calculated as

(8) $$E\left[P_{I_{agg}}\right]=E\left[\sum_{i=1}^{N}P_{I_{i}}\right]=E\left[\sum_{i=1}^{N}Ah_{i}r_{i}^{-\alpha }\right]=AE\left[N\right]E\left[h_{i}\right]E\left[r_{i}^{-\alpha }\right]\;.$$

If the average channel gain $E\left[h_{i}\right]$ is assumed to be 1, i.e., normalized to unity with $E\left[h_{i}\right]=1$, substituting (4) and (7) into (8) we obtain:

(9) $$E\left[P_{I_{agg}}\right]\;=\;A\lambda_{I}{\left.\frac{t{\left(\frac{t^{2}}{L^{2}}+1\right)}^{\frac{\alpha }{2}}{}_{2}F_{1}\left(\frac{1}{2},\frac{\alpha }{2};\frac{3}{2};-\frac{t^{2}}{L^{2}}\right)}{{\left(t^{2}+L^{2}\right)}^{\frac{\alpha }{2}}}\right|}_{d_{s}}^{d_{e}}\;.$$

The variance value of the interference power of the *i*th interfering radar is given by

(10) $$Var\left[P_{I_{i}}\right]=E\left[P_{I_{i}}^{2}\right]-{\left(E\left[P_{I_{i}}\right]\right)}^{2}\;=\;A^{2}E\left[h_{i}^{2}\right]E\left[r_{i}^{-2\alpha }\right]-A^{2}{\left(E\left[h_{i}\right]\right)}^{2}{\left(E\left[r_{i}^{-\alpha }\right]\right)}^{2}\;,$$

where the term $E\left[r_{i}^{-2\alpha }\right]$ can be computed by Formula (7) replacing the variable α with 2α. We consider a wide range of fading environments, such as no-fading, Rayleigh fading and Nakagami fading cases. The channel fading gain is set to 1 in the no-fading case. After normalization, $h_{i}$ follows an exponential distribution or a gamma distribution in the case of Rayleigh or Nakagami fading, namely $h_{i}∼\mathrm{Exp}\left(1\right)$ or $h_{i}∼\mathrm{Gamma}\left(m_{L},\frac{1}{m_{L}}\right)$. Then, the second moment of the channel fading gain is represented by

(11) $$E\left[h_{i}^{2}\right]=\left\{\begin{array}{cc} 2, & Rayleigh\;fading\\ \frac{m_{L}+1}{m_{L}}, & Nakagami\;fading \end{array}\right.\;,$$

where $m_{L}$ is the Nakagami fading index for LOS, which is supposed to be integer in our analysis. Especially, when $m_{L}=1$, the Nakagami fading is equivalent to the Rayleigh fading case.

Similarly, the variance value of the aggregated interference power is:

(12) $$Var\left[P_{I_{agg}}\right]=E\left[{P_{I_{agg}}}^{2}\right]-{\left(E\left[P_{I_{agg}}\right]\right)}^{2}\;.$$

The second moment of the total interference is given by

(13) $$E\left[{P_{I_{agg}}}^{2}\right]=E\left[{\left(\sum_{i=1}^{N}Ah_{i}{r_{i}}^{-\alpha }\right)}^{2}\right]=E\left[N^{2}\right]{\left(E\left[P_{I_{i}}\right]\right)}^{2}+E\left[N\right]Var\left[P_{I_{i}}\right]\;.$$

Finally, the variance is formulated as

(14) $$Var\left[P_{I_{agg}}\right]=Var\left[N\right]{\left(E\left[P_{I_{i}}\right]\right)}^{2}+E\left[N\right]Var\left[P_{I_{i}}\right]\;.$$

Substituting (4), (6) and (10) into (14), we can obtain the variance value of the aggregated interference power for the typical radar receiver under three fading channel cases with any path-loss exponent.

### 3.2. Interference with Gaussian Attenuation Directional Radiation Pattern

In this part, we employ a widely used directional antenna model [28] to calculate the aggregated interference power, which has a main lobe of Gaussian form and a constant level of side lobes. With this model, the directional antenna gain is given by

(15) Gθp=G0e−4ln2θpθpθ−3dBθ−3dB2,0≤θp<θmlθml22Gsl,else,

where $\theta_{p}$ is the angle between the LOS direction of interferer–victim pair and the center line of the transmitter/receiver’s beam. $\theta_{ml}$ and $\theta_{-3dB}$ represent the main lobe width and antenna half power beamwidth (HPBW), respectively. If not specified, the beamwidth θ in our study generally refers to the HPBW. $G_{0}$ denotes the maximum antenna gain expressed by (16) in units of decibel (dB):

(16) G0=10ln1.6162sinθ−3dBθ−3dB222.

The constant side lobe gain can be obtained by $G_{sl}=-0.4111\cdot \ln\left(\theta_{-3dB}\right)-10.597$, which causes little effect on interference compared to the main lobe, especially when the beam is very narrow. Therefore, in the proposed interference model, we do not explore the effects of side lobes on the extent of automotive radar interference. Note that $\theta_{p}=0$ signifies the perfect beam alignment of a interferer–victim pair which is the worst scenario leading to a higher interference level.

For simplicity, we take the no-fading case with the path-loss exponent of 2, for example, to characterize the interference statistics based on the above directional antenna model. The analysis methodology can also be applied to different channel fading cases with any path-loss exponent. The interference power of the individual interfering radar with the Gaussian decaying antenna gain is rewritten as

(17) $$P_{I}=\frac{P_{t}{G_{0}}^{2}\lambda^{2}}{{\left(4\pi \right)}^{2}}e^{-8\ln2{\left(\frac{\theta_{p}}{\theta_{-3dB}}\right)}^{2}}r^{-2}=Ae^{a\theta_{p}^{2}}r^{-2}\;,$$

where the subscript *i* is omitted for notational simplicity and the parameter a=−8ln2−8ln2θ−3dB2θ−3dB2. We can calculate the mean interference of the interferers to the typical radar as follows:

(18) $$\begin{array}{cc} E\left[P_{I_{agg}}\right] & \;=\;E\left[\sum_{i=1}^{N}Ae^{a\theta_{p}^{2}}r^{-2}\right]\;=\;AE\left[N\right]E\left[e^{a\theta_{p}^{2}}r^{-2}\right]=AE\left[N\right]\cdot \int_{d_{s}}^{d_{e}}e^{a\theta_{p}^{2}}r^{-2}f_{D}\left(t\right)dt\\ \; & =\frac{A\lambda_{I}}{L}\int_{\arctan\frac{L}{d_{e}}}^{\frac{\theta }{2}}e^{a\theta_{p}^{2}}d\theta_{p}={\left.\frac{A\lambda_{I}}{L}\cdot \frac{\sqrt{\pi }}{2\sqrt{-a}}\mathrm{erf}\left(\sqrt{-a}\;\theta_{p}\right)\right|}_{\arctan\frac{L}{d_{e}}}^{\frac{\theta }{2}} \end{array}\;,$$

in which $\mathrm{erf}\left(\cdot \right)$ is the error function, the horizontal distance d=LLtanθptanθp and the Euclidean distance r=LLsinθpsinθp between a certain interferer and the victim. When the interfering vehicle is located at $d_{s}$, the corresponding directional angle θp=θθ22. The angle θp=arctanLLdede while the interfering vehicle is located at $d_{e}$. In the worst case, the upper bound of the distance is infinite while the lower bound of the corresponding angle is 0. We obtain a compact closed-form expression of mean interference power in this limit case, written as follows:

(19) $$E_{w}\left[P_{I_{agg}}\right]\;=\;\frac{\lambda_{I}A}{L}\int_{0}^{\frac{\theta }{2}}e^{a\theta_{p}^{2}}d\theta_{p}\;=\;\frac{\lambda_{I}A}{L}\frac{\sqrt{\pi }}{2\sqrt{-a}}\mathrm{erf}\left(\frac{\theta \sqrt{-a}}{2}\right)\;.$$

Similarly, the variance value of total interference power considering the above directional radiation pattern is obtained by -4.6cm0cm

(20) $$Var\left[P_{I_{agg}}\right]=Var\left[N\right]A^{2}{\left(E\left[e^{a\theta_{p}^{2}}r^{-2}\right]\right)}^{2}\;+\;E\left[N\right]A^{2}Var\left[e^{a\theta_{p}^{2}}r^{-2}\right]\;=\;A^{2}E\left[N\right]E\left[{\left(e^{a\theta_{p}^{2}}r^{-2}\right)}^{2}\right]\;,$$

where the second moment $E\left[{\left(e^{a\theta_{p}^{2}}r^{-2}\right)}^{2}\right]$ is calculated by

(21) $$\begin{array}{cc} E\left[{\left(e^{a\theta_{p}^{2}}r^{-2}\right)}^{2}\right] & \;=\;\frac{1}{d_{e}-d_{s}}\frac{1}{L^{3}}\int_{\arctan\frac{L}{d_{e}}}^{\frac{\theta }{2}}e^{2a\theta_{p}^{2}}\sin^{2}\left(\theta_{p}\right)d\theta_{p}=\frac{1}{d_{e}-d_{s}}\frac{1}{L^{3}}\int_{\arctan\frac{L}{d_{e}}}^{\frac{\theta }{2}}e^{2a\theta_{p}^{2}}\theta_{p}^{2}d\theta_{p}\\ \; & \;=\;{\left.\frac{1}{d_{e}-d_{s}}\frac{1}{L^{3}}\left(\frac{\theta_{p}e^{2a\theta_{p}^{2}}}{4a}-\frac{\sqrt{2\pi }\;\mathrm{erfi}\left(\sqrt{2a}\theta_{p}\right)}{16\;a^{\frac{3}{2}}}\right)\right|}_{\arctan\frac{L}{d_{e}}}^{\frac{\theta }{2}} \end{array}\;.$$

The beamwidth θ of FR is very narrow and the directional angle θp<θθ22, hence we adopt the approximation $\sin\left(\theta_{p}\right)\approx \theta_{p}$ in Formula (21). $\mathrm{erfi}\left(\cdot \right)$ is the imaginary error function. The variance of the aggregated interference power in the limit case is given by

(22) $$Var_{w}\left[P_{I_{agg}}\right]\;=\;\frac{\lambda_{I}A^{2}}{L^{3}}\left(\frac{\frac{\theta }{2}e^{2a{\left(\frac{\theta }{2}\right)}^{2}}}{4a}-\frac{\sqrt{2\pi }\mathrm{erfi}\left(\frac{\theta \sqrt{2a}}{2}\right)}{16\;a^{\frac{3}{2}}}\right)\;.$$

### 3.3. Interference Considering Blockage

The signals of mmWave automotive radars are blocked, which is mainly caused by the vehicles running on the road. We consider these blocking vehicles modeled as a 1-D PPP with intensity $\lambda_{B}$ in each lane. We consider the complete blockage case under opposite direction two-lane traffic scenario, where the signal beamwidth θ of vehicular FR is blocked by the vehicle in front under the same lane with the interfering vehicle. As shown in Figure 2, the radiation beam of the interferer V4 is obstructed by V3, and more significantly, the V3 is both a blocker and a interferer.

The signal from an interferer is completely blocked, if at least one blockage is presented and located at a distance of less than $l_{0}$ to the given interferer. The probability that there is no vehicle appearing within the distance of $l_{0}$ is $p_{NB}=\mathrm{Pr}\left\{N_{P}\left(l_{0}\right)=0\right\}=\frac{{\left[\lambda_{B}l_{0}\right]}^{0}}{0!}e^{-\lambda_{B}l_{0}}=e^{-\lambda_{B}l_{0}}$, hence, the blocking probability is given by

(23) $$p_{B}=1-\mathrm{Pr}\left\{N_{P}\left(l_{0}\right)=0\right\}=1-e^{-\lambda_{B}l_{0}}\;,$$

where the distance l0=wc2tanθθ22 with $w_{c}$ representing the width of vehicle.

The number of the interferers *N* follows Poisson distribution with density $\lambda_{I}$. The interferer not being blocked is termed active interferer. The number of active interferers *K* is expressed as

(24) $$K=X_{I_{1}}+X_{I_{2}}+\cdots +X_{I_{i}}+\cdots +X_{I_{N}}\;,$$

where $X_{I_{i}}$ is a binary random variable which is set to 1 indicating that the *i*th interferer is not blocked with probability $p_{NB}$, which is otherwise equal to 0 to signify the interferer blocked with the probability $p_{B}$. The probability generating function (PGF) of $X_{I_{i}}$ is calculated by

(25) $$G_{X_{I_{i}}}\left(z\right)=p_{B}+\left(1-p_{B}\right)z=1-e^{-\lambda_{B}l_{0}}+e^{-\lambda_{B}l_{0}}z\;.$$

Suppose whether each interferer is blocked or not is independent of each other, and the PGF of *K* is derived as

(26) $$G_{K}\left(z\right)=E\left(z^{\sum_{i=1}^{N}X_{I_{i}}}\right)=G_{N}\left(G_{X_{I_{i}}}\left(z\right)\right)=e^{\lambda_{I}\left(d_{e}-d_{s}\right)\left(G_{X_{I_{i}}}\left(z\right)-1\right)}=e^{\lambda_{I}\left(d_{e}-d_{s}\right)e^{-\lambda_{B}l_{0}}\left(z-1\right)}\;.$$

The result of Formula (26) shows that the number of active interferers *K* is also a Poisson random variable with parameter $\lambda_{I}\left(d_{e}-d_{s}\right)e^{-\lambda_{B}l_{0}}$. In addition, the mean and variance of *K* in the interval $\left[d_{s},d_{e}\right]$ are $E\left[K\right]=D\left[K\right]=\lambda_{I}\left(d_{e}-d_{s}\right)e^{-\lambda_{B}l_{0}}=\lambda_{I}\left(d_{e}-d_{s}\right)\left(1-p_{B}\right)$. Replacing $E\left[N\right]$ and $D\left[N\right]$ with $E\left[K\right]$ in (18) and (20), we can therefore obtain the statistical characteristics of the aggregated interference power in the two-lane scenario considering the blockage and employing directional antenna radiation with Gaussian waveform. Similarly, the levels of interference in the limited case can also be evaluated the same way.

## 4. Interference Analysis Extension to Multiple-Lane Scenarios

In this section, we extend the interference analysis to multiple lanes scenario, where the mean value of the total interference for the victim vehicle at any lane is derived firstly without consideration of the blockage, and then the blockage probability is formulated based on the proposed blocking model to assess the impact of blockage on interference.

### 4.1. Without Considering the Blockage

We assume there are *n* lanes in the opposite direction and *m* lanes in same direction with that of the victim vehicle. From the innermost lane to the outermost lane, the interfering lanes are marked as 1,2,⋯,j,⋯,n, and the lanes of another driving direction are marked as 1,2,⋯,i,⋯,m. We utilize an independent 1-D PPP to model the geometry distribution of vehicles in the lanes of each direction with same density ρ.

We do not consider the blockage impact on automotive radar signal in this part. The interference is caused by the interfering FRs on vehicles from the lanes 1,⋯,j,⋯,n, therefore the mean interference received at the FR of the typical vehicle in lane 1 is computed by -4.6cm0cm

(27) $$\begin{array}{cc} \; & E\left[P_{I_{agg}}^{1}\right]=E\left[P_{I_{agg}}^{11}+\cdots +P_{I_{agg}}^{1j}+\cdots +P_{I_{agg}}^{1n}\right]=E\left[P_{I_{agg}}^{11}\right]+\cdots +E\left[P_{I_{agg}}^{1j}\right]+\cdots +E\left[P_{I_{agg}}^{1n}\right]\\ \; & E\left[P_{I_{agg}}^{1j}\right]=\frac{AE\left[N_{j}\right]}{jL\left(d_{e_{j}}-d_{s_{j}}\right)}\int_{\arctan\frac{jL}{d_{e_{j}}}}^{\frac{\theta }{2}}e^{a\theta_{p}^{2}}d\theta_{p} \end{array}\;,$$

where $N_{j}$ denotes the number of the interferers in the *j*th interfering lane with mean $E\left[N_{j}\right]$ in the interval $\left[d_{s_{j}},d_{e_{j}}\right]$, i.e., $E\left[N_{j}\right]=\lambda_{I}\left(d_{e_{j}}-d_{s_{j}}\right),\;j=1,2,\cdots ,n$. The upper bound of angle θθ22 corresponds to a different lower bound of distance $d_{s_{j}}$ for different interfering lanes. The maximum distances of interference for these lanes are labeled as $d_{e_{j}}$ relative to the lower bound of angle. We set $d_{e_{j}}=jd_{e}$ and then the mean interference of the victim in lane 1 is rewritten as

(28) $$E\left[P_{I_{\mathrm{agg}}}^{1}\right]=\sum_{j=1}^{n}\frac{A\lambda_{I}}{jL}\int_{\arctan\frac{L}{d_{e}}}^{\frac{\theta }{2}}e^{a\theta_{p}^{2}}d\theta_{p}=\sum_{j=1}^{n}\frac{A\lambda_{I}}{jL}{\left.\cdot \frac{\sqrt{\pi }}{2\sqrt{-a}}\mathrm{erf}\left(\sqrt{-a}\;\theta_{p}\right)\right|}_{\arctan\frac{L}{d_{e}}}^{\frac{\theta }{2}}\;.$$

Consequently, the mean interference of the typical vehicle at lane *i* can be solved by Formula (29) in the limit case.

(29) $$E_{w}\left[P_{I_{agg}}^{i}\right]=\sum_{j=1}^{n}\frac{A\lambda_{I}}{\left(i+j-1\right)L}\cdot \frac{\sqrt{\pi }}{2\sqrt{-a}}\mathrm{erf}\left(\sqrt{-a}\;\frac{\theta }{2}\right)\;,$$

where *n* is the number of interfering lanes and i∈1,2,⋯,m. The calculation of variance can also be implemented according to the above method and in reference to (20), which will not be detailed here.

We can explore the interference level of mmWave automotive radars including BR and SR as a function of vehicle density and signal beamwidth, as well as the radar on-vehicle mounting location. Suppose that the typical vehicle is located at lane *i*, the specific interference situations are as follows.

(1) Interference to SRL from SRRs and SRLs: the interfering SRRs are on vehicles from the lanes 1,⋯,i−1, and the interfering SRLs are on vehicles from the lanes 1,⋯,j,⋯,n;
(2) Interference to SRR from SRLs: the interfering SRLs are on vehicles from the lanes i+1,⋯,m;
(3) Interference to BRL from BRLs: the interfering BRLs are on vehicles from the lanes 1,⋯,j,⋯,n.

The above analysis is on account of the prerequisite that different types of radars operate at a different frequency. The characterization of the interference extents for SR and BR can be obtained according to the solution method of FR.

### 4.2. Considering the Blockage

We consider both the complete and partial blockage cases in the multi-lane scenario and model the blocking vehicles on each lane as 1-D PPP with the same line intensity $\lambda_{B}$. Due to FR is mainly interfered with by the FRs on oncoming vehicles in opposite lanes, the blockage situations can be described as follows.

(1) Complete blockage: the interfering signal is completely blocked by the vehicle ahead moving in the same lane as the given interferer. In each interfering lane exists the complete blockage case, where the blockage probability can be calculated by

(30) pB1=1−e−λBl0,l0=wc2tanθθ22.

(2) Partial blockage: assume the interfering vehicle in lane *j* and the typical vehicle in lane *i*, the transmit beam of the interferer–victim pair is partially obstructed by the blockages including the vehicles from the lanes 1,⋯,j−1 and the vehicles from the lanes 1,⋯,i−1. Thus, the probability of partial blockage for the *k*th interferer in lane *j* blocked by the vehicles in one of the blockage lanes is denoted as

(31) $$p_{B2}=\frac{E\left[N_{B}\right]\cdot l_{c}}{l_{B_{ij}}^{k}}=\frac{\lambda_{B}l_{B_{ij}}^{k}\cdot l_{c}}{l_{B_{ij}}^{k}}=\lambda_{B}l_{c}\;,$$

where $l_{c}$ is the length of vehicle, $N_{B}$ is the number of blockages within the blockage range $l_{B_{ij}}^{k}$ with mean $E\left[N_{B}\right]=\lambda_{B}l_{B_{ij}}^{k}$. The blockage range is given by

(32) $$l_{B_{ij}}^{k}\;=\;\left(i+j-1\right)L\left[\tan\left(\frac{\pi }{2}-\theta_{p_{ij}}^{k}\right)\;-\;\tan\left(\frac{\pi }{2}-\frac{\theta }{2}\right)\right]\;,$$

in which *i* and *j* are the lane marks where the victim and the interferer are located, respectively, the directional angle $\theta_{p_{ij}}^{k}=\arctan\left(\frac{\left(i+j-1\right)L}{d_{ij}^{k}}\right),\;k=1,2,\cdots ,N_{j}$, $d_{ij}^{k}$ is the horizontal distance between the *k*th interferer and the victim.

**Figure 3:**
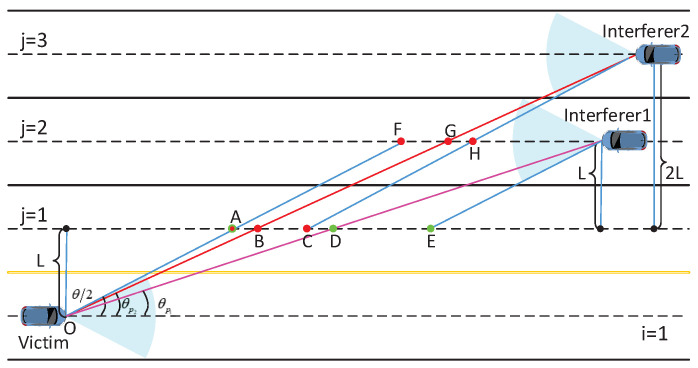
An example of partial blockage where the victim is in lane 1 and interferers in lanes 2 and 3.

Consequently, the partial blockage probability of the *k*th interferer on the *j*th lane being blocked by the vehicles on its left lanes is $\left(j-1\right)p_{B2}+\left(i-1\right)p_{B2}$. Note that the partial blocking is not considered in a two-lane scenario, as is the case for i=1,j=1 which does not apply to (32). Figure 3 illustrates an example of partial blockage where the victim is located at the origin on lane 1 and interferers are on lanes 2 and 3. The signal beam of the interferer 2 and victim pair is partially blocked by the vehicles within the blockage range $\bar{\mathrm{FH}}$ and $\bar{\mathrm{AC}}$ ($\bar{\mathrm{FH}}=\bar{\mathrm{AC}}$), and the signal beam of the interferer 1 and victim pair is partially blocked by the vehicles with the blockage range $\bar{\mathrm{AE}}$. This blockage range can be acquired by (32).

Finally, we obtain the blockage probability of the interferers on lane *j* expressed as $P_{B}^{j}=\left\lfloor p_{B1}+\left(i+j-2\right)p_{B2}\;,1\right\rfloor$. As shown in Formula (27), most of interference power at the typical receiver is created by the closest interferers. In other words, the level of the interference is usually attenuated with the distance between the interferer and victim. Conversely, the farther the interferer is from the victim, the greater the probability of being blocked. Ultimately, the interferer can be almost completely blocked if it is far away enough from the typical receiver.

Based on the analysis result of Formula (26), the average number of the active interferers in the *j*th interfering lane is $E\left[K_{j}\right]=\lambda_{I}\left(d_{e_{j}}-d_{s_{j}}\right)\left(1-p_{B}^{j}\right)$. We obtain the mean interference power of the typical radar under the considering blockage situation, substituting $E\left[K_{j}\right]$ for $E\left[N_{j}\right]$ in (27).

## 5. Automotive Radar Performance

The performance of mmWave automotive radars is likely to be limited by interference and noise. We leveraged the ranging and detection success probability as the metric to evaluate radar performance. The successful ranging probability $p_{s}$ of automotive radar is defined as the complementary cumulative distribution function (CCDF) of SINR evaluated at a threshold η which is:

(33) $$p_{s}\;=\;\mathrm{Pr}\left\{SINR\geq \eta \right\}\;=\;\mathrm{Pr}\left\{\frac{P_{echo}}{P_{I_{agg}}+P_{N}}\geq \eta \right\}\;.$$

In order to calculate the above probability, we rearrange the parameters in (33) and obtained the following expression:

(34) $$p_{s}\;=\;\mathrm{Pr}\left\{P_{I_{agg}}\leq \frac{P_{echo}}{\eta }\;-\;P_{N}\right\}\;=\;F_{P_{I_{agg}}}\left(\frac{P_{echo}}{\eta }\;-\;P_{N}\right)\;,$$

where $F_{P_{I_{agg}}}$ denotes the CDF of the aggregated interference power at the typical radar. $P_{N}=k_{B}T_{0}B_{0}F_{0}$ represents the power of the additive noise, in which $k_{B}$ is Boltzmann’s constant, $T_{0}$ is the standard temperature, $B_{0}$ is the equivalent noise bandwidth of FR and $F_{0}$ is the system loss factor.

There are two ways to obtain the CDF of the aggregated interference power. In the first approach, using Gil-Pelaez’s inversion formula [29]:

(35) FPIaggx=12−1π∫0∞Ime−isxφPIaggs1sds=12−1π∫0∞Ime−isxMPIaggis1sds,

and making the following substitution:

(36) $$M_{P_{I_{agg}}}\left(s\right)\;=\;G_{N}\left(M_{P_{I}}\left(s\right)\right)\;=\;e^{\lambda_{I}\left(d_{e}-d_{e}\right)\left(M_{P_{I}}\left(s\right)-1\right)}=\exp\left(\lambda_{I}\int_{d_{s}}^{d_{e}}\left(e^{sAe^{a\theta_{p}^{2}}r^{-2}}-1\right)dt\right)\;,$$

the probability $p_{s}$ can be directly acquired. $\varphi_{P_{I_{agg}}}\left(s\right)$ is the characteristic function (CF), and the moment generating function (MGF) $M_{P_{I_{agg}}}\left(s\right)$ is represented under without considering blockage and employing the directional antenna with Gaussian decaying gain, in which the MGF of the individual interference power can also be expand to $M_{P_{I}}\left(s\right)=\sum_{k=0}^{\infty }\frac{s^{k}E\left[P_{I}^{k}\right]}{k!}$. The approach is usually used to find the CDF of a random variable *X* on account of its MGF $M_{X}\left(s\right)$ which is easy to solve. However, this integral is very difficult to achieve while the complex expression of $M_{P_{I_{agg}}}\left(s\right)$ is substituted into (35).

In what follows, we describe another approach to find $F_{P_{I_{agg}}}$ and obtain the radar ranging success probability. According to the statistical analysis for each total interference power and their histogram, we assume that the aggregated interference subjects to a gamma distribution with shape parameter μ and scale parameter ν given as

(37) $$\mu =\frac{{\left(E\left[P_{I_{agg}}\right]\right)}^{2}}{Var\left[P_{I_{agg}}\right]},\;\;\nu =\frac{Var\left[P_{I_{agg}}\right]}{E\left[P_{I_{agg}}\right]}\;.$$

Consequently, the closed-form expression of the automotive radar ranging success probability for the path-loss exponent of 2 is expressed by

(38) $$p_{s}=\frac{1}{\Gamma \left(\mu \right)}\Upsilon \left(\mu ,\frac{\frac{A_{0}d_{0}^{-4}}{\eta }-P_{N}}{\upsilon }\right)\;,$$

where $\Gamma \left(\cdot \right)$ is the gamma function, and $\Upsilon \left(\cdot ,\cdot \right)$ is the lower incomplete gamma function.

## 6. Simulation Results

In this section, simulations are carried out to verify the derived analytical results presented in this paper. We first explore the interference levels of automotive radar in terms of interfering vehicle density, directional antenna pattern, channel fading, path-loss exponent, as well as blockage effect. Subsequently, we validate the proposed interference distribution from the empirical model and the histogram obtained by numerical statistics. At last, the successful ranging probability $p_{s}$ of the automotive radar is plotted in different scenarios.

We consider a 1000 m-long straight segment for the two-lane scenario, whereas the length of the road segment is related to the lane location of the typical vehicle for a multi-lane scenario, e.g., the victim on lane 1, de1 = 1000 m and the victim on lane 2, de2 = 2000 m. In order to obtain the statistic of interference, 100,000 Monte Carlo trails are conducted for plotting each line in simulations. Simulation parameter settings are listed in Table 1, and our simulated setup is described as follows. We generate the number and locations of interfering vehicles randomly following a 1-D thinning PPP with a density of $\lambda_{I}=\varepsilon \rho$. That is, the first step is to obtain the random number *N* of interferers obeying Poisson distribution with intensity $\lambda_{I}$, and then to generate the locations of these interferers uniformly distributed over the range $\left(0,d_{e}\right)$ according to the random number *N*. The channel fading and the path-loss exponent are simulated following the description in Section 3.1. In blocking modeling, we set the length and width of vehicle as equal to 4 and 2, namely $l_{c}=4$ m and $w_{c}=2$ m. The number and locations of blockages are randomly generated following 1-D PPP with intensity $\lambda_{B}$. The RCS $\sigma_{c}$ of the detected target is assumed to be a constant although it could be fluctuating.

### 6.1. Radar Interference

**Figure 4:**
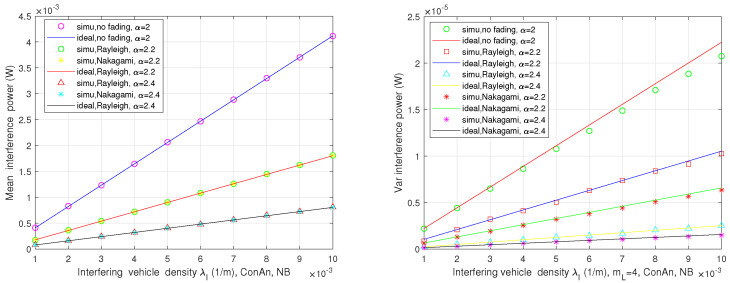
Mean and variance of interference power vs. interfering vehicle density in two-lane scenario.

**Figure 5:**
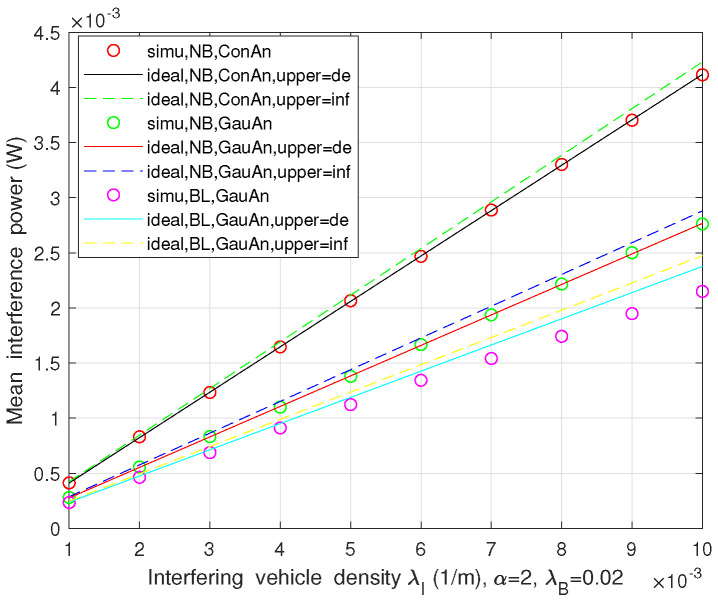
The mean interference power vs. interfering vehicle density in three conditions.

**Figure 6:**
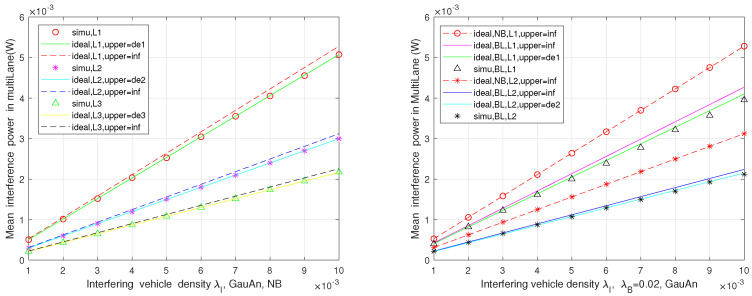
The mean interference power with and without blockage in multi-lane scenarios.

In this part, the interference levels of automotive FR in both two-lane and multi-lane scenarios are presented, which are affected by different parameters. As it was illustrated in Figure 4, Figure 5 and Figure 6, Monte Carlo simulations (simu) match basically well with the theoretical analysis (ideal).

Figure 4 demonstrates the mean and variance values of the aggregated interference power at the typical receiver in the two-lane situation using the directional antenna with constant gain (ConAn) for different channel fading cases. The plot indicates that the simulation results are consistent with the closed-form expressions in (9) and (14), where the blockage is not taken into account (NB) and $d_{e}=1000$ m, ε=0.1. Obviously, the mean interference power increases with the interfering vehicle density. In addition, the greater path-loss exponent α brings the smaller mean interference. When the channel fading gain is normalized, the mean interference powers for Rayleigh fading and Nakagami fading cases with the same α value cannot be distinguished. Although the normalized fading channel has no effect on the mean interference, it has an impact on the interference variance. As can be seen from this figure, the variance in the no-fading case with α=2 is larger than that in Rayleigh and Nakagami fading cases with α slightly exceeding 2. In addition, the variance value for the Rayleigh fading channel fluctuates more greatly than the corresponding value in the Nakagami fading case with the same path-loss exponent.

Figure 5 shows the mean interference power at FR with varying the interfering vehicle density in three cases: (1) no blocking (NB) with ConAn; (2) NB employing the directional antenna with Gaussian decaying gain (GauAn); and (3) considering the blockage (BL) with GauAn. It can be observed that the simulated interference power is linearly proportional to the interfering vehicle density. Moreover, the mean interference with the constant gain antenna is significantly larger than the ones utilizing the antenna with non-constant gain, which indicates that the consideration of the directional antenna radiation gain related to the overlapping beam of interferer–victim pair can understand the levels of interference more accurately. The result also obeys the fact that the interference power considering the blockage case is lower than the cases without blocking. We plot the theoretical analysis for mean interference with upper bound (inf) in the latter two cases, referring to (19), whereas EwPIagg=λIAθλIAθ2L2L according to Campbell’s theorem in ConAn, NB case.

The mean interference at the typical vehicle in multi-lane scenarios is presented in Figure 6 for both no blockage and the considering blockage. We formulate the lanes per driving direction (LPD) to 3, i.e., n=m=3, and the victim vehicle is arranged in lane 1 (L1) or lane 2 (L2) or lane 3 (L3). The plot on the left side shows that the interference power of the victim vehicle in lane 1 is the largest, followed by those in lane 2 and lane 3, since the closer the victim is to the interferers, the greater the interference will be. In the plot on the right side, it is obvious to see that the more serious interference occurs for the victim in lane 1 rather than in lane 2, resulting from the blockages appearing for the latter. This is the mean interference level affected by the distance between the victim and interferers, interfering with the vehicle density, as well as the blockage, in addition to the directional antenna radiation pattern and the channel fading. Similarly, the limit cases of interference for each scenario are plotted.

### 6.2. Model Validation

In the proposed analytical method of radar performance, the critical assumption is the gamma distribution of total interference. To validate the method, we simulate the deployment scenarios, described in the above section. We first obtain the interference statistic histograms from simulated data, and then based on the histograms, we compare the analytical and empirical PDFs for different ρ in both two-lane and multi-lane scenarios. Moreover, we plot the CDF comparison between the gamma model and the simulated data.

**Figure 7:**
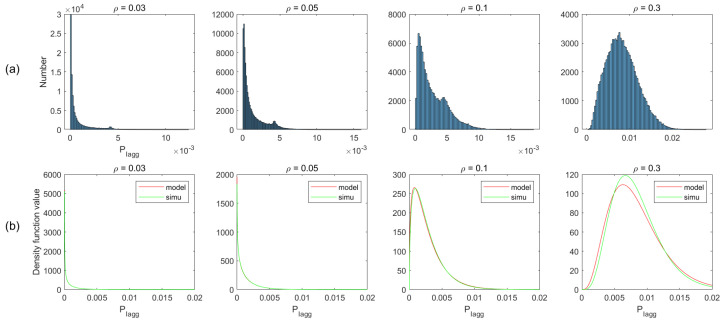
(**a**) Non-normalized histogram; (**b**) The gamma PDF at each of the values in $P_{I_{agg}}$ using the corresponding shape parameters in μ and scale parameters in υ, for two-lane scenario without considering blockage. These two parameters are obtained from the simulation data (simu) or theoretical derivation (model), and ε=0.1, α=2, GauAn.

**Figure 8:**
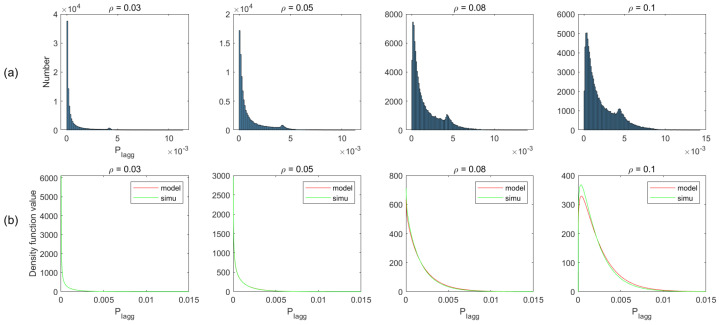
Non-normalized histogram and probability density function (PDF) of total interference in two-lane scenario with considering blockage.

**Figure 9:**
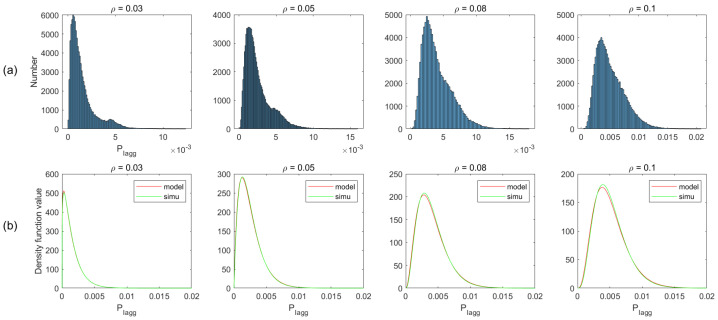
Non-normalized histogram and probability density function (PDF) of total interference in multi-lane scenario without considering blockage.

**Figure 10:**
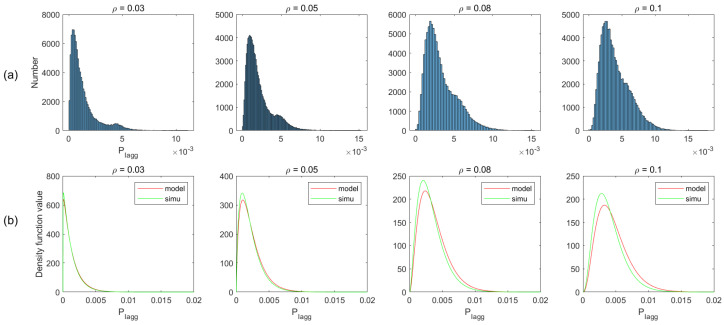
Non-normalized histogram and probability density function (PDF) of total interference in multi-lane scenario with considering blockage.

Figure 7a plots the histogram of aggregated interference from the simulation of different vehicle density ρ, and the corresponding PDFs in the gamma model and simulated data are shown in Figure 7b. As can be observed from this figure, the simulation is basically consistent with the model, apart from some existing bumps. We ignore the bumps and do not consider it as a rule for modeling the interference distribution. The reason is that when the vehicle density is higher, the bump is gradually weakened, even disappearing (e.g., ρ=0.3). This is especially the case for multi-lane scenarios, where the bumps are insignificant due to the increase in the number of interfering vehicles. Therefore, we assume that the distribution of the aggregated interference power roughly obeys gamma distribution. Similar matching has been observed for other conditions, as shown in Figure 8, Figure 9 and Figure 10.

**Figure 11:**
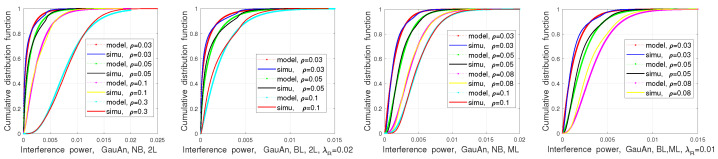
Cumulative distribution function (CDF) of total interference in two-lane (2L) and multi-lane (ML) scenarios.

Figure 11 compares CDFs of the aggregated interference for a different ρ in the above four conditions. Similarly to PDFs of interference, we see matching between model and simulations. These results conclude that both PDFs and CDFs of interference are roughly captured by the proposed model. Thus, in the next section, we evaluate the radar performance based on the proposed model.

### 6.3. Radar Performance

We numerically evaluate the successful ranging probability $p_{s}$ vs. ranging distance $d_{0}$, with different vehicle density ρ in the two-lane and multi-lane scenarios employing a directional antenna with a Gaussian decaying gain and the considering blockage or not.

**Figure 12:**
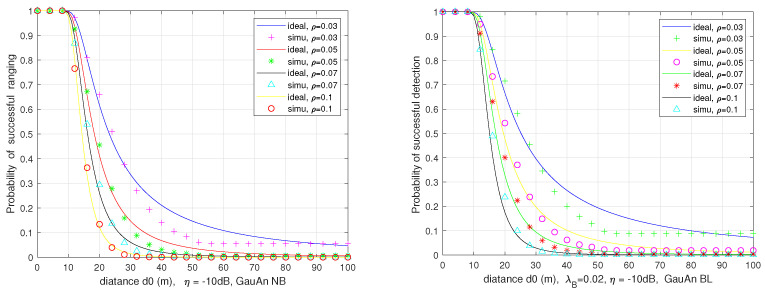
The successful ranging probability vs. the ranging distance in two-lane scenario with and without considering blockage.

Figure 12 plots the relation between $p_{s}$ and $d_{0}$ in (38) for the no-fading case with α=2 in the two-lane scenario. The figure also shows the approximate consistency of the theoretical analysis and Monte Carlo simulations. We can see that $p_{s}$ declines with the ranging distance $d_{0}$, which is due to the fact that the power of desired echoes diminishes with the fourth power of range, resulting in the degradation of SINR. It also can be observed that $p_{s}$ rises as ρ declines, because the victim radar receiver suffers from less interference signal with smaller interfering intensity.

**Figure 13:**
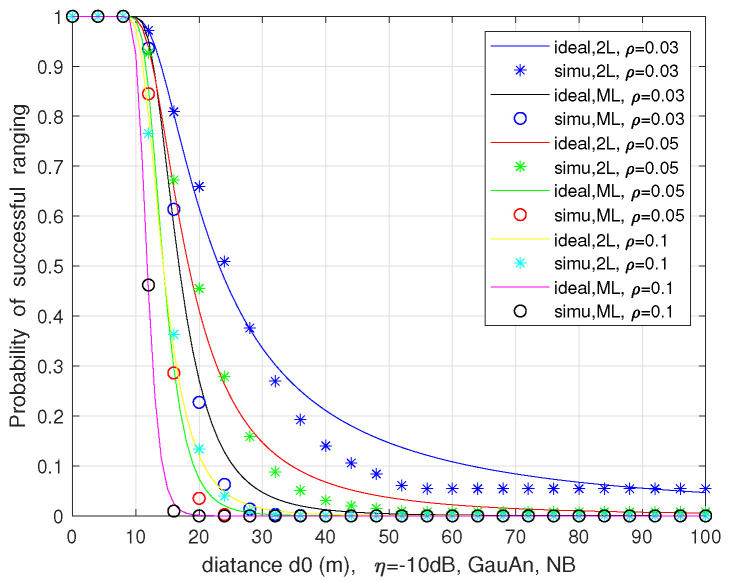
Comparison the probability of successful ranging in two-lane (2L) and multi-lane (ML) scenarios without considering blockage.

**Figure 14:**
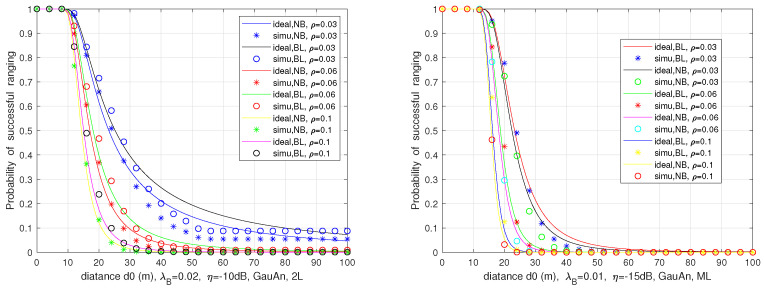
Comparison the probability of successful ranging in two-lane or multi-lane scenario with and without considering blockage.

Figure 13 provides a comparison of success ranging probability in both two-lane and multi-lane scenarios without considering blockage. Compared with the two-lane, the radar performance decreases at the same set of input parameters in the multi-lane scenario. The reason is that the stronger interference level is experienced by the victim radar on the multi-lane case. The probability of successful ranging in the two-lane scenario with and without blockage is compared in Figure 14 on the left side. As expected, the non-considered blockage leads to an increase in interference level, and thus declines the automotive radar ranging performance. Similar results are also presented in the plot on the right side for multi-lane scenario, where the gap becomes narrow due to the smaller blockage probability.

## 7. Conclusions

In this paper, we developed an analytical method for the mmWave automotive radar interference using stochastic geometry. Directional antennas with constant and Gaussian decaying gain were included in the analysis. Furthermore, the effect of blockages on mmWave radar signals was also taken into consideration. We first derived the closed-form expressions for the mean and variance of interference power at the typical radar receiver in two-lane scenarios and then extended the work to multi-lane ones. Based on the interference characterization, we formulated the radar performance evaluation method utilizing the successful ranging probability. Finally, we verified the proposed interference analysis method by simulation in the presence of a Poisson field of interferers and blockages. The results show that considering the directional antenna gain related with the directional angle between the victim and interferer pair is more reasonable for the interference analysis. In addition, considering blocking factors makes the interference analysis more accurate. As expected, the interference level with blockage is more tally with the actual one. This paper provides the insights to understand the interference levels in automotive radars, which is necessary to design the effective mitigating or avoiding an algorithm in the future.

## Figures and Tables

**Table 1:** Simulation parameter settings.

Parameter	Value	Parameter	Value
$P_{t}$	10 dBm	$f_{c}$	76.5 GHz
$\sigma_{c}$	10 dBsm [30]	*c*	$3\times 10^{8}$ m/s
θ	$15^{\circ }$ [25]	$T_{0}$	290 K
*L*	3.5 m [31]	$B_{0}$	25 kHz [25]
$k_{B}$	$1.38\times 10^{-23}$ J/K	$F_{0}$	15 dB

## Data Availability

Not applicable.
